# Actin depolymerization by Latrunculin B can either suppress or promote root gravitropism, depending on the developmental stages in Arabidopsis

**DOI:** 10.1080/15592324.2025.2581654

**Published:** 2025-11-09

**Authors:** Ai Chen, Qianqian Wang, Xianyong Sheng

**Affiliations:** College of Life Sciences, Capital Normal University, Beijing, People's Republic of China

**Keywords:** Arabidopsis, root, gravitropism, actin, developmental stages

## Abstract

Root gravitropism enables plants to optimize water and nutrient uptake, with actin filaments playing a key regulatory role. However, the effects of F-actin depolymerization on gravitropism have been inconsistent. Here, we show that actin depolymerization impacts root gravitropism in a developmentally dependent manner. In newly germinated roots, weak statolith constraint by actin means depolymerization does not significantly enhance statolith sedimentation but inhibits cell elongation on the upper root side, reducing gravitropic bending. In mature roots, stronger statolith constraint allows actin depolymerization to promote statolith sedimentation and inhibit cell elongation on the lower side, thus accelerating root bending. These findings provide new perspectives for a deeper understanding of the mechanisms underlying root gravitropism.

Root gravitropism constitutes a key adaptive mechanism that has evolved in plants during their long-term adaptation to terrestrial environments. This trait not only optimises the efficiency of water and mineral nutrient acquisition essential for plant growth and development, but also reinforces the mechanical support of aerial organs, thereby markedly enhancing the overall structural stability of the plant.^1-3^ Amyloplasts are widely regarded as the primary sensors for gravity perception in plants.^4-6^ However, the mechanism by which the physical signal of gravitational potential energy from amyloplasts is transduced into intracellular biological signals remains elusive. Actin filaments are among the most important intracellular structures, extensively involved in regulating a range of critical biological processes such as organelle movement, signal transduction, and polarised cell growth.^7-9^ It has been proposed that, under gravitational stimulation, the sedimentation of amyloplasts may induce changes in the tension of the actin cytoskeleton network, thereby activating mechanosensitive ion channels on the plasma membrane or causing deformation of the endoplasmic reticulum or vacuolar membranes, which in turn initiates downstream gravitropism-related signal transduction pathways.^7^^,^^10^^,^^11^ Surprisingly, disruption of F-actin by pharmacological or genetic approaches has been reported to enhance, inhibit, or have no effect on root gravitropism.^12-19^ To date, a comprehensive mechanistic explanation that reconciles these apparently contradictory findings is still lacking.

Recent studies have shown that nascent primary roots respond significantly faster to gravity than mature roots,^20^ which raises the possibility that F-actin depolymerisation may differentially affect root gravitropism at distinct developmental stages. To test this hypothesis, the effects of the actin depolymerisation via Latrunculin B on gravitropic bending were compared between newly germinated (1.5-day-old) and mature (5-day-old) primary roots. Our results showed that when seedlings were horizontally reorientated on standard 1/2 MS medium (containing 1% sucrose and 1% agar), 1.5-day-old and 5-day-old primary roots exhibited gravitropic bending rates of approximately 25.36 ± 2.06°/h and 10.84 ± 1.36°/h, respectively. After three hours, the bending angles reached 76.09 ± 6.17° and 32.51 ± 4.08°, respectively (Figure 1a, c, e). Statistical analysis indicated that, under normal medium, 1.5-day-old young roots and 5-day-old mature roots elongated vertically downward at approximately 92.1 ± 13.03 μm/h and 167.45 ± 18.6 μm/h. Given that the growth rate of 1.5-day-old roots was markedly lower than that of 5-day-old roots (Figure 1f), yet their gravitropic bending rate was higher (Figure 1e), our data are consistent with previous reports indicating that the difference in gravitropic bending rates between young and mature roots primarily results from the greater sensitivity of young roots to gravitational stimuli, rather than from differences in growth rate per se.^20^ Surprisingly, when 1.5-day-old roots were incubated with 1 μM Latrunculin B for 1 h prior to reorientation, their average bending rate over the subsequent 3 h was approximately 20.37 ± 2.99°/h, equivalent to only 80.31% of untreated controls (Figure 1a, b, e). In contrast, 5-day-old roots subjected to the same treatment bent at 16.07 ± 2.49°/h, reflecting an increase of 48.27% compared to their controls (Figure 1c–e). Meanwhile, the growth rates of 1.5-day-old and 5-day-old roots decreased to 55.55 ± 10.98 μm/h and 111.55 ± 18.65 μm/h, respectively (Figure 1f). These findings indicate that the impact of actin depolymerisation on root gravitropism varies with developmental stage, potentially inhibiting, promoting, or having no impact depending on root age.

**Figure 1.**
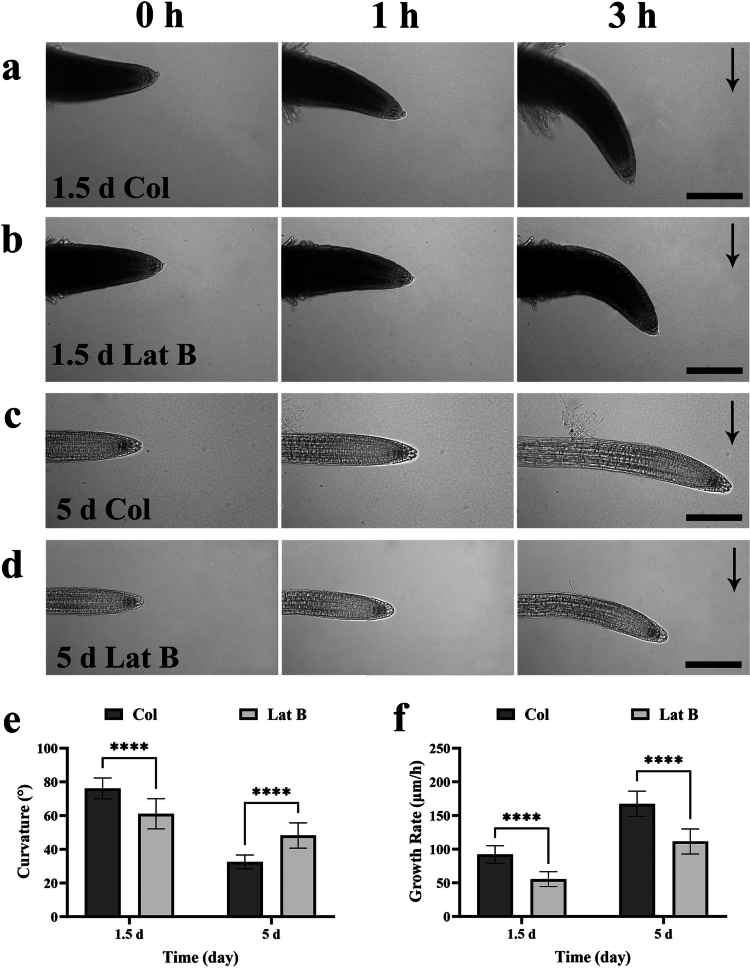
Latrunculin B inhibits gravitropism in newly germinated roots but promotes it in mature roots. Seeds of *Arabidopsis thaliana* (Col-0) were grown on standard medium for 1.5 days (newly germinated roots) and 5 days (mature roots) were treated with 0 or 1 μM Latrunculin B for 1 h. The seedlings were then rotated 90° and placed on a vertical stage microscope for an additional 3 h. Root growth rate and gravitropic bending rate were analysed using ImageJ soft. (a) 1.5 d control roots. (b) 1.5 d roots treated with 1 μM Latrunculin B. (c) 5 d control roots. (d) 5 d roots treated with 1 μM Latrunculin B. (e) Quantification of gravibending angles after 3 h 90° rotation in control and Latrunculin B-treated groups for both newly germinated and mature roots (n = 24). (f) Comparison of growth rates between control and Lat B-treated groups in newly germinated and mature roots (n = 20). Data are presented as mean ± standard deviation. Two-way ANOVA was performed; *p < 0.05, **p < 0.01, ****p < 0.0001. Black arrows indicate the direction of gravity. Scale bar:100 μm.

In light of the starch*-*statolith hypothesis, amyloplasts in columella cells play an essential role in gravity sensing.^21^^,^^22^ This hypothesis is supported by substantial experimental evidence.^5^^,^^23^^,^^24^ Nonetheless, some studies have reported that starch-deficient mutants, such as *pgm*-*1* and *adg1*-*1*, are still capable of exhibiting a gravitropic response, although they require significantly longer periods of gravistimulation.^25^^,^^26^ Therefore, it has been proposed that plastids themselves, rather than starch granules, perform an essential function in gravity perception.^5^^,^^25^^,^^27^^,^^28^ This implies that plastids in the columella cells of these mutants are still capable of sedimenting in response to gravitational stimulation. To test this hypothesis, *pgm-1* mutants expressing the mEosFP*-*tagged plastid marker protein tpFNR were used in subsequent experiments. The observations indicate that in vertically downward*-*oriented *pgm-1* roots, plastids sediment at the bottom of columella cells in the direction of gravity (Figure 2b). After rotating the roots by 180°, these plastids clearly exhibit a tendency to sediment along the direction of gravity (Figure 2c), demonstrating that starch-deficient plastids indeed retain a certain capacity for gravity-directed sedimentation. However, compared to the wild type, plastid movement in *pgm-1* columella cells was markedly slower. Specifically, after 4 h, although the plastids had moved away from their original positions, they had not yet reached the new gravity-facing side of the cells (Figure 2c). By this time, the root tips had already exhibited significant gravitropic bending (Figure 2a), consistent with previous reports.^26^ In contrast, in *pgm-1* roots treated with 1 μM Latrunculin B for 1 h followed by 180° reorientation (Figure 2e), amyloplasts in the columella cells sedimented at a markedly accelerated rate, with most reaching the new gravity–facing side of the cells within 3 h (Figure 2f). These results suggest that actin filament depolymerisation is likely to promote amyloplast sedimentation in columella cells, thereby facilitating the gravitropic response (Figure 2d).

**Figure 2.**
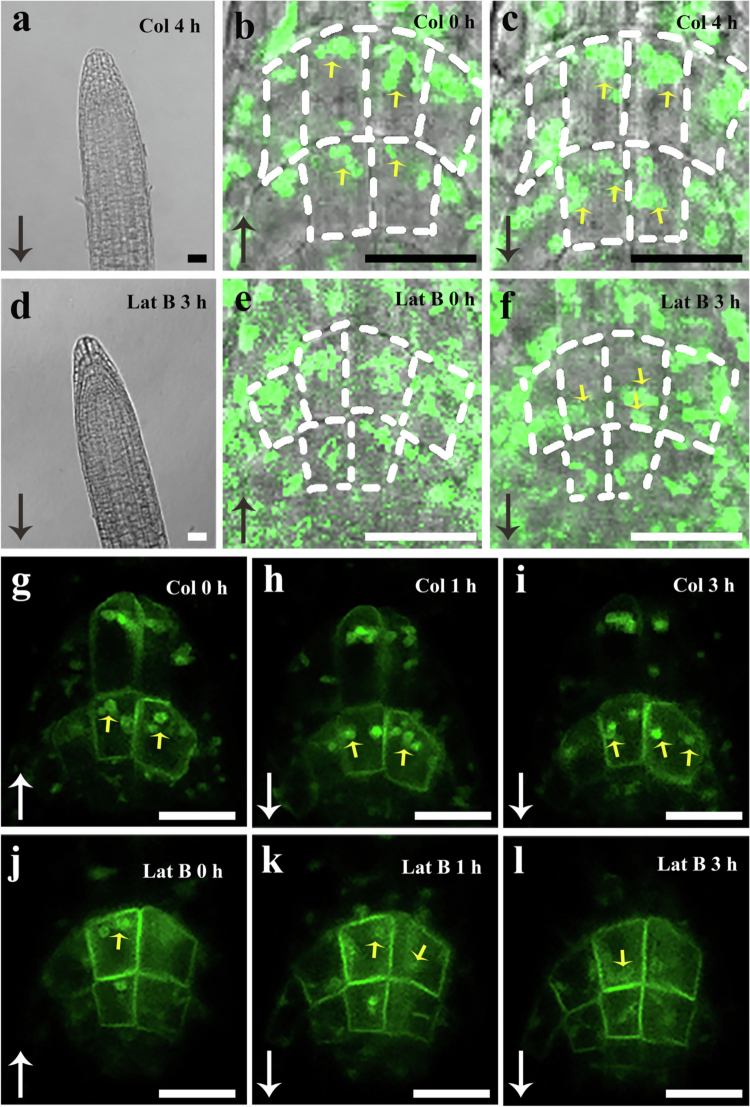
Latrunculin B promotes gravitropic sedimentation of plastids in the columella cells of *pgm-1.* Homozygous tpFNR:mEosFP × *pgm-1* (a–f) or LAZY-GFP × *pgm-1* (g–l) hybrid seedlings were grown on standard medium for 5 d and then treated with 0 or 1 μM Latrunculin B for 1 h. Subsequently, control and 1 μM Latrunculin B-treated seedlings were rotated 180° and incubated for an additional 3–4 h. The gravitropic localisation of plastids in columella cells was observed using a Leica SP8 laser scanning confocal microscope. Yellow arrows indicate plastid positions. Long arrows denote the direction of gravity. Experiments were independently repeated more than three times. Scale bar: 20 μm.

The relocalization of LAZY family proteins to the plasma membrane of columella cells is considered to play an essential role in root gravitropism.^4^^,^^6^ Previous studies have suggested that gravitational stimulation may trigger phosphorylation of LAZY family proteins and promote their enrichment on the surface of amyloplasts through interactions with TOC proteins (Translocon at the Outer Envelope Membrane of Chloroplasts). As amyloplasts sediment along the gravity vector, LAZY family proteins are consequently guided to relocalize to the new lower plasma membrane of columella cells.^4^^,^^6^ In this study, we generated a genetic cross between LAZY3-GFP and *pgm-1* to examine the spatial distribution of LAZY family proteins in starch-deficient mutants. Confocal observation revealed that LAZY3 proteins were localised both at the plasma membrane and on plastids, which allowed clear visualisation of cell outline and the spatial relationship between the plasma membrane and plastids (Figure g–l). Our results confirmed that treatment with Latrunculin B promoted plastid sedimentation and the subsequent relocalization of LAZY3 proteins to the plasma membrane (Figure j–l). These findings align with the view that LAZY family proteins redistribute concurrently with amyloplast sedimentation after gravistimulation.^4^^,^^6^ However, after a 4-h 180° reorientation, mutant roots exhibited a pronounced gravitropic curvature. However, LAZY3-GFP-labelled plastids failed to reach the plasma membrane on the new gravity-facing side and remained suspended within the cytoplasm (Figure i). It is highly likely that plastid removal from the plasma membrane, rather than their arrival at the new bottom membrane, may represent a more critical signalling event in transducing the gravity stimulus into intracellular signalling.^29^ Additionally, under non-stimulated conditions, only a small proportion of plastids closely apposed to the plasma membrane. In contrast, following gravistimulation, all plastids traverse the cytoplasm along the gravity vector and inevitably interact with various cellular structures, including the endoplasmic reticulum, vacuoles, and, most notably, the cytoskeleton. Therefore, the possibility that signal transduction is triggered by plastid sedimentation within the cytoplasm, rather than exclusively by their interactions with the plasma membrane, should not be ruled out.

Previous studies have reported that the minimal effect of Latrunculin B on root gravitropism is due to the incomplete development of the actin cytoskeleton in columella cells of newly emerged flax roots.^15^ Therefore, we hypothesised that developmental differences in the ability of actin filaments to constrain amyloplasts could underlie the stage-specific responses of roots to actin-depolymerising agents. To test this hypothesis, the organisation of actin cytoskeleton and the gravity-induced sedimentation of amyloplasts in columella cells from both nascent and mature roots of transgenic Arabidopsis expressing GFP-fABD2 were analysed using confocal microscopy.^17^ The results showed that, in 5-day-old roots, strong actin filament bundles were widely observed in the cytoplasm, particularly surrounding the amyloplasts in columella cells (Figure 3c). In contrast, in 1.5-day-old roots, actin filaments appeared thinner, shorter, and more diffuse (Figure 3a). Consistent with our previous report,^17^ treatment with 100 nM Latrunculin B for 1 h moderately depolymerised actin filaments in both 1.5-day-old and 5-day-old roots (Figure 3c, d). Time-series analysis revealed that amyloplasts in normally cultured root caps did not exhibit saltation, unlike those in stem endodermal cells where a subset underwent rapid long–distance transport along actin filaments.^30^^,^^31^ Instead, they displayed active oscillatory motion, particularly in columella cells adjacent to the quiescent centre (Supplementary Video 1). And this behaviour was almost completely abolished following Latrunculin B treatment (Supplementary Video 2). A similar phenomenon was observed for vacuoles in the elongation zone cells, where their active movement was also markedly suppressed by Latrunculin B (Supplementary Videos 3 and 4). These results indirectly indicate that Latrunculin B treatment effectively disrupts the structural integrity and functional dynamics of microfilaments. Interestingly, in 5-day-old roots, upon vertical reorientation, amyloplasts in the innermost one to two layers of columella cells sedimented to the gravity–facing side of the cells within 5-8 minutes, whereas most of amyloplasts in the third layers of columella cells from the inside exhibited no significant gravity-induced sedimentation even after 3 h (Figure 3g, h). This is consistent with a previous report suggesting that the innermost one to two layers of columella cells play a more critical role in gravity signal perception.^32^ Moreover, when roots were treated with Latrunculin B for 1 h and then reoriented vertically, amyloplasts, including those in the third layer of columella cells, were found to sediment toward the new gravity-facing bottom within the subsequent 3 h following gravistimulation (Figure 3k, l). These findings indicate that the actin cytoskeleton network regulates the positioning and dynamics of amyloplasts. In contrast, insufficient development of the actin cytoskeleton in young root caps imposes relatively weak restraint on amyloplasts, allowing greater mobility and faster sedimentation under gravity (Figure 3e, f). Accordingly, this may also explain why actin depolymerisation exerts little effect on amyloplast sedimentation in 1.5-day-old roots (Figure 3i, j).

**Figure 3.**
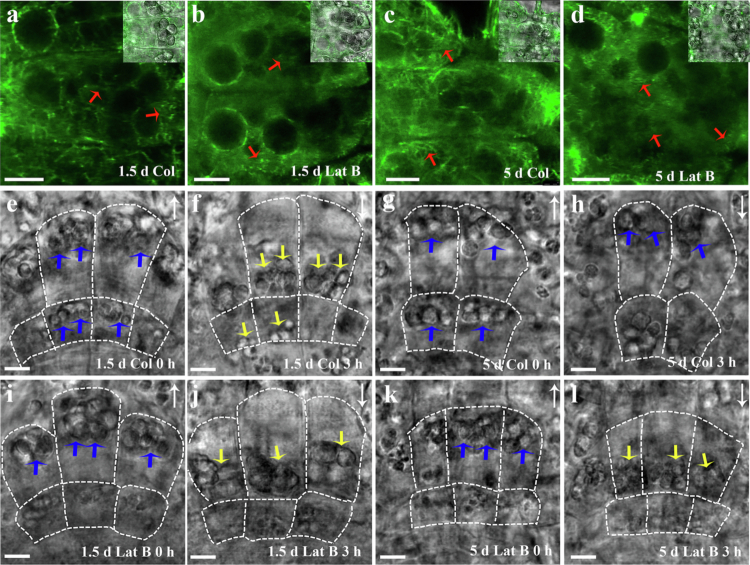
Latrunculin B enhances plastid sedimentation in mature root columella cells but has no significant effect in newly emerged roots. Seeds of transgenic *Arabidopsis thaliana* expressing GFP-fABD2 were grown on standard medium for 1.5 days or 5 days, then treated with 0 nM or 100 nM Latrunculin B for 1 h. Seedlings were subsequently rotated 180° and incubated for an additional 3 h. The morphology of actin microfilaments and the distribution of amyloplasts in columella cells were observed using a Leica SP8 laser scanning confocal microscope. (a–d): GFP fluorescence images illustrating the morphology and distribution of the microfilament cytoskeleton in control and Latrunculin B–treated roots, respectively. (e–l): The distribution of amyloplasts within columella cells. Yellow or blue arrows indicate the positions of amyloplasts. White long arrows denote the direction of gravity. Scale bar: 5 μm.

The bending growth of plant organs is driven by asymmetric growth between cells on opposite sides.^33^^,^^34^ In roots, this process occurs at the root tips, with the transition and elongation zones making the major contribution.^17^ Using GFP-Lit6a, a transgenic Arabidopsis line expressing green fluorescent protein on the plasma membrane,^17^ we found that, under normal medium conditions, although cell growth in the elongation zone proceeds more slowly in 1.5-day-old roots compared to 5-day-old roots, the difference in growth rates between the upper and lower sides of the elongation zone following gravitropic stimulation is greater in 1.5-day-old roots than in 5-day-old roots (Figure 4a, c, e, g). Furthermore, Latrunculin B treatment markedly inhibits cell elongation on both the upper and lower sides of the elongation zone, regardless of root age (Figure 4b, d, f, h). To further elucidate the differential effects of Latrunculin B treatment on gravitropic bending in young and mature roots, we comparatively analysed the changes in growth rates of epidermal cells on the upper and lower sides–from the root cap through the elongation zone–following gravistimulation. Statistical analysis demonstrated that, when newly emerged roots grown on standard medium are placed horizontally, gravitational stimulation leads to a marked increase in the growth rate on the upper side, reaching approximately 116 ± 8.8 μm/h. In contrast, growth on the lower side is strongly inhibited, with a rate of only about 43.22 ± 11.09 μm/h, which is approximately 37.2% of that on the upper side. As a result, there is a pronounced difference in growth rates between the two sides, amounting to 72.78 ± 10.54 μm/h (Figure 4i, k). In comparison, when mature roots are placed horizontally, the growth rate on the lower side decreases by 27.44%, whereas the growth rate on the upper side shows no significant difference relative to the vertical orientation (Figure 4j, k). Therefore, although mature roots exhibit a higher growth rate than newly emerged roots, growth rate difference after horizontal placement is only 19.94 ± 4.96 μm/h. Consequently, the gravitropic bending rate of mature roots is lower than that of newly emerged roots, as mentioned above (Figure 1a, c). In contrast, Latrunculin B treatment exerted opposite effects on cell elongation rates between the upper and lower sides of horizontally placed newly emerged and mature roots. In newly emerged roots, treatment with Latrunculin B significantly inhibited cell elongation on the upper side, while mildly suppressing elongation on the lower side, leading to a reduced difference in growth rate between the two sides (48.41 ± 9.79 μm/h) compared to the control (Figure 4i, k). In mature roots, however, Latrunculin B markedly inhibited cell elongation on both the upper and lower sides, with the inhibition being particularly pronounced on the lower side (a decrease of 40.75%). As a result, the growth rate difference between the two sides increased to 27.83 ± 4.76 μm/h (Figure 4j, k). This contrasting response may be attributed to the fact that, under gravistimulation, cell elongation on the lower side of newly emerged roots is already strongly suppressed, leaving limited potential for further inhibition by Latrunculin B. In contrast, cell elongation on the upper side is accelerated and therefore more susceptible to microfilament disruption, which reduces the difference in growth rates between the two sides and ultimately results in a slower gravitropic bending rate following Latrunculin B treatment. In mature roots, however, both the upper and lower sides maintain relatively high cell elongation rates under gravistimulation. As a result, Latrunculin B treatment markedly suppresses cell elongation on both sides, especially the lower side, thereby enhancing the growth rate difference and consequently accelerating the gravitropic bending rate.

**Figure 4.**
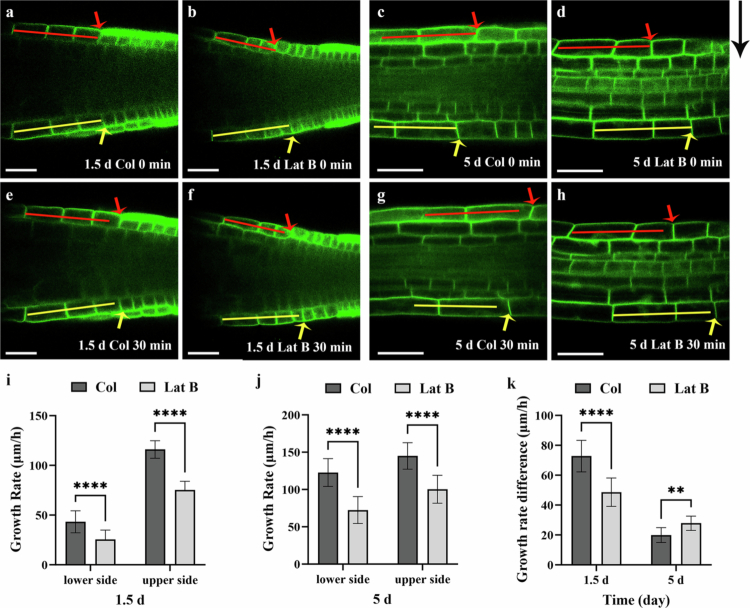
Latrunculin B decreases growth rate differences during gravitropic bending in newly germinated roots but increases them in mature roots. (a–h): Seeds of transgenic *Arabidopsis thaliana* expressing GFP-Lti6a were grown on standard medium for 1.5 days and 5 days. Seedlings were then treated with 0 or 1 μM Latrunculin B for 1 h, followed by a 90° rotation and imaging on a vertical-stage confocal microscope for an additional 30 minutes. The lengths of the red (or yellow) lines in the first row are identical to those of the corresponding red (or yellow) lines in the second row. Coloured arrows indicate the boundaries of the cells at the right end of each line; the distance between each arrow and the adjacent line in the second row represents the increase in length of the corresponding elongation zone cell after 30 minutes of gravistimulation. The long black arrow denotes the direction of gravity. Scale bar: 50 μm. (i) and (j): Growth rates of upper and lower side epidermal cells from the root cap to the elongation zone in 1.5-day-old and 5-day-old control and Latrunculin B treated roots, respectively, after 3 hours of gravistimulation (*n* = 18). (k): The differential growth rates of upper and lower side epidermal cells from the root cap to the elongation zone in 1.5-day-old and 5-day-old control and Latrunculin B treated roots, respectively, after 3 hours of gravistimulation (*n* = 18). Data are expressed as mean ± standard deviation. Two-way ANOVA was performed; *p < 0.05, **p < 0.01, ****p < 0.0001.

In conclusion, this study reveals that the effect of actin depolymerisation on root gravitropism varies depending on the developmental stage of the root. In newly germinated roots, the constraint of statoliths by actin filaments in columella cells is relatively weak; actin depolymerisation does not significantly promote the gravitropic sedimentation of statoliths, but markedly inhibits the elongation of cells on the upper side of the root, resulting in a reduced rate of gravitropic bending. In contrast, in more mature roots, the constraint of statoliths by actin filaments in columella cells is stronger; actin depolymerisation significantly promotes the gravitropic sedimentation of statoliths and more prominently inhibits the elongation of cells on the lower side of the root, thereby accelerating root bending. These findings provide new perspectives for a deeper understanding of the mechanisms underlying root gravitropism.

## Supplementary Material

Supplementary Material Supplementary_video_1_Col_cap

Supplementary Material Supplementary_video_2_Lat_B_cap

Supplementary Material Supplementary_video_3_Col_Vacuole

Supplementary Material Supplementary_video_4_Lat_B_Vacuole
